# Modulation of Innate Immunity by G-CSF and Inflammatory Response by LBPK95A Improves the Outcome of Sepsis in a Rat Model

**DOI:** 10.1155/2018/6085095

**Published:** 2018-11-07

**Authors:** Haoshu Fang, Chuanfeng Hua, Stefanie Weiss, Anding Liu, Wenhui Cheng, Ralf Claus, Jürgen Rödel, Olaf Dirsch, Uta Dahmen

**Affiliations:** ^1^Department of Pathophysiology, Anhui Medical University, Hefei, China; ^2^Experimental Transplantation Surgery, Department of General, Visceral and Vascular Surgery, Jena University Hospital, Jena, Germany; ^3^Laboratory Animal Research Center, College of Basic Medical Sciences, Anhui Medical University, Hefei, China; ^4^Experimental Medicine Center, Tongji Hospital, Tongji Medical College, Huazhong University of Science and Technology, Wuhan, China; ^5^Integrated Research and Treatment Center, Center for Sepsis Control and Care (CSCC), Jena University Hospital, Jena, Germany; ^6^Institute of Medical Microbiology, Jena University Hospital, Jena, Germany; ^7^Institute for Pathology, Hospital of Chemnitz, Chemnitz, Germany

## Abstract

**Introduction:**

Sepsis is the primary cause of death from infection. We wanted to improve the outcome of sepsis by stimulating innate immunity in combination with modulating the severity of inflammatory responses in rats.

**Method:**

Sepsis was induced by the injection of feces suspension (control). A 5-day course of G-CSF treatment was given before the septic insult (G-CSF). The inflammatory response was decreased using various doses of the LPS-blocking peptide LBPK95A (5 mg/kg = 100% Combi group, 0.5 mg/kg = 10% Combi group, and 0.05 mg/kg = 1% Combi group). Survival rates were observed. Bacterial clearance, neutrophil infiltration, tissue damage, and the induction of hepatic and systemic inflammatory responses were determined 2 h and 12 h after the septic insult.

**Results:**

High-dose LBPK95A (100% Combi) reduced the survival rate to 10%, whereas low-dose LBPK95A (10% and 1% Combi) increased the survival rates to 50% and 80%, respectively. The survival rates inversely correlated with multiorgan damage as indicated by the serum levels of ALT and urea. G-CSF treatment increased the white blood cell counts, hepatic neutrophil infiltration, and bacterial clearance in the liver, lung, and blood. The blockade of the LPS-LBP interaction decreased neutrophil infiltration, led to increased white blood cell count, and decreased hepatic neutrophil infiltration, irrespective of dose. However, bacterial clearance improved in the 1% and 10% Combi groups but worsened in the 100% Combi group. G-CSF increased TNF-*α* and IL-6 levels. Irrespective of dose, the blockade of the LPS-LBP interaction was associated with low systemic cytokine levels and delayed increases in hepatic TNF-*α* and IL-6 mRNA expression. The delayed increase in cytokines was associated with the phosphorylation of STAT3 and AKT.

**Conclusion:**

Our results revealed that increasing innate immunity by G-CSF pretreatment and decreasing inflammatory responses using LBPK95A improved the survival rates in a rat sepsis model and could be a novel strategy to treat sepsis.

## 1. Introduction

Sepsis is defined as the overwhelming reaction to the invasion of microorganisms and their components. The organism mounts an innate immune response to eliminate pathogens. Sepsis is frequently associated with increased blood levels of endotoxin [1]. Endotoxin leads to dose-dependent inflammatory responses, ultimately resulting in SIRS, endotoxin shock, and death. The clinical picture of sepsis can be dominated by the bacteria-host interaction, the inflammatory response, or a combination of both. The activation of lymphocyte contributes to bacterial clearance but simultaneously triggers the inflammatory response that in turn causes systemic injury.

Despite years of research, optimal strategies that specifically target the aggressive immune response that characterizes sepsis are not yet available [2]. Modulation of innate immunity to increase bacterial clearance and decrease the inflammatory response is a novel strategy to treat sepsis. Recently, there have been various experimental approaches to treat sepsis by strengthening the host's immune response to invading microorganisms [3].

Granulocyte colony-stimulating factor (G-CSF) is a hematopoietic growth factor that is released after infection and increases the number and function of polymorphonuclear neutrophils (PMNs) [4]. In the intact organism, activated PMNs are key components in host defense during acute bacterial infection [5–7], promoting the elimination of bacteria. Therefore, the stimulation of neutrophils is an appealing approach to the treatment of infections [8]. G-CSF is beneficial for early survival during sepsis. In a clinical trial, G-CSF was applied prophylactically in patients undergoing major surgery, resulting in a clear tendency towards lowering the rate of postoperative septic complications [9]. However, previous experimental studies regarding therapy for sepsis by means of G-CSF returned conflicting results [10, 11].

In previous experiments, we observed that the injection of G-CSF leads to an increase in lipopolysaccharide binding protein (LBP) expression [12]. LBP is named for its ability to bind to LPS. The binding of LBP to LPS is the first step in the mechanism of LPS recognition by the innate immune system. Binding between LBP and LPS activates the inflammatory response [13] and leads to increased bacterial clearance [14, 15].

Taken together, one reason for the observed conflicting results of G-CSF-treatment could be an inappropriate balance of the putatively beneficial effect of LBP-mediated bacterial clearance and the detrimental effect of LPS-sensitization in the course of sepsis development. Therefore, we hypothesized that the dynamic balance between LBP-mediated LPS-sensitization and bacterial clearance was decisive for the therapeutic success of G-CSF-induced modulation of innate immunity in sepsis.

## 2. Materials and Methods

### 2.1. Animals

Male inbred Lewis rats (300 ± 50 g; Charles River, Sulzfeld, Germany) were used in this study. All animals were housed under standard animal care conditions and had access to water and rat chow ad libitum. Male rats were used to avoid hormonal fluctuations. The permission for animal experiments was given by the “Thüringer Landesamt für Verbraucherschutz” (AZ: 2226840402026/13).

Animals were allowed to adapt to laboratory conditions for at least seven days. All procedures were carried out according to German Animal Welfare Legislation and were performed under inhalation anesthesia with 3% isoflurane (Sigma Delta, London, UK) complemented by the injection of the analgesic drug Torbugesic Vet (5 *μ*g/kg).

### 2.2. Experimental Design

To induce sepsis, rats were injected intraperitoneally with human stool suspension (control group). Innate immunity was enhanced by pretreating rats with G-CSF (G-CSF group, 100 *μ*g/kg/day, subcutaneous injection, Ratiopharm, Breda, Netherlands) for 5 days before septic insult. The inflammatory response was blocked by interfering with the LPS-LBP interaction using an LBP inhibitory peptide, LBPK95A (sequence: RVQGRWKVRASFFK, the peptide was synthesized in-house using an Fmoc standard procedure on an ABI 433A peptide synthesizer), by intraperitoneal injection [16]. The toxic effect of LBPK95A was detected by administration of LBPK95A (5 mg/kg, intraperitoneal) or 0.9% NaCl to rats, respectively (*n* = 3). The rats were sacrificed 24 h after injection, and the serum ALT was measured. To modulate the severity of inflammatory response, three doses—5 mg/kg (100% Combi group), 0.5 mg/kg (10% Combi group), and 0.05 mg/kg (1% Combi group) of LBPK95A—were selected and were injected simultaneously with the septic insult after G-CSF pretreatment. According to the previous results [14], the treatment of LBPK95A only did not show any protective effect after septic insults; therefore, in the present study, the LBPK95A + sepsis group was not included.

For survival analysis, rat activity was monitored every 3 h up to 72 hours (*n* = 10 per group). In the kinetic experiment, the rats were sacrificed at 2 h (*n* = 6) and 12 h (*n* = 6). Tissue injury, bacterial clearance, neutrophil infiltration, activation of signal pathways, and induction of local and systemic inflammatory responses at 2 h and 12 h after septic insult were analyzed.

### 2.3. Sepsis Model

A sepsis model mimicking the complexity of the clinical situation was used. Therefore, peritoneal contamination and infection (PCI) was performed in rats, using a human feces suspension comprised of several bacteria, induced as described by Gonnert et al. [17]: 3 *μ*l/g body weight stool suspension, diluted 1 : 4 in saline, was injected intraperitoneally (ip) into the right lower quadrant of the abdomen with a 21-gauge cannula.

### 2.4. Monitoring and Sampling

At 2 h and 12 h after septic insult, the rats were sacrificed under 3% isoflurane anesthesia. The ascites were collected, and the volume was recorded. Blood was taken from the vena cava for blood count, liver enzyme, and cytokine analysis. The liver and lung were sampled for histological evaluation. Liver tissue samples were stored in −80°C until use in gene and protein expression studies.

### 2.5. Histological Staining

Liver and lung tissue was fixed in 4.5% buffered formalin for at least 24 h. Paraffin embedding was performed, and sections (4 *μ*m) were cut and stained with hematoxylin-eosin (HE). Slides were digitalized using a virtual slide scanner (Hamamatsu Electronic Press Co., Ltd, Iwata, Japan). Histological evaluation was performed according to a standardized semiquantitative scoring system [16].

### 2.6. Naphthol-AS-D-chloroacetate Esterase (ASDCL) Staining

Neutrophil infiltration into the liver was evaluated by ASDCL staining in liver tissues, as reported previously [16]. After staining, slides were digitalized using the virtual slide scanner, and 10 high-power fields (HPF) pictures at a magnification of 400× were randomly selected for analysis. ASDCL-stained positive neutrophils were counted manually. The results were calculated as the number of positive staining cells per HPF.

### 2.7. Clinical Chemistry

To assess the liver and kidney damage, serum ALT and urea were measured using an automated Chemical Analyzer (F. Abbott Architect®cil6200).

### 2.8. Enzyme-Linked Immunosorbent Assay (ELISA)

For the analysis of serum TNF-*α* and IL-6 levels, commercially available enzyme-linked immunosorbent assay kits were used (R&D systems, Minneapolis, US). The procedures were performed according to the manufacturer's suggestions. Measurements of the ELISA were performed in 96-well polystyrene plates using an SLT Spectra ELISA plate reader at 450 nm.

### 2.9. Electrophoresis and Western Blot (WB)

Fifteen micrograms of total liver lysate protein or serum were loaded per well and were separated on 12% gels by sodium dodecyl sulfate-polyacrylamide gel electrophoresis, followed by Western blotting and staining with rabbit anti-phospho-AKT (Ser 473) (1 : 1000, Cell Signaling Technology), rabbit anti-AKT (1 : 1000, Cell Signaling Technology), rabbit anti-phospho-Stat3 (Tyr 705) (1 : 2000, Cell Signaling Technology), rabbit anti-STAT3 (1 : 2000, Cell Signaling Technology), orrabbit anti-caspase 3 (1,1000, Cell Signaling Technology). Signals were detected with Lumi-Light Western blot substrate (Thermo, Waltham, US) and exposure to X-ray film (GE Healthcare, Buckinghamshire, UK).

### 2.10. Quantitative Polymerase Chain Reaction (qPCR)

RNA was isolated from liver tissues using the RNeasy kit (Qiagen, Hilden, Germany), and the procedure was performed according to the manufacturer's instructions. The concentration of RNA was quantified by the relation of absorbance at 260 nm to absorbance at 280 nm. Total RNA (2–5 *μ*g) was reverse transcribed using the First-Strand cDNA synthesis kit (Invitrogen, Carlsbad, USA). Real-time PCR was carried out in an M3000P QPCR System (Stratagene, La Jolla, USA), using the Brilliant probe-based QPCR Master Mix kit (Agilent, Santa Clara, USA), probes (Universal Probe Library), and primers: IL-6: CCTGGAGTTTGTGAAGAACAACT, GGAAGTTGGGGTAGGAAGGA, and probe #106; TNF-*α*: TGAACTTCGGGGTGATCG, GGGCTTGTCACTCGAGTTTT, and probe #63; hypoxanthine guanine phosphoribosyltransferase (HPRT): GACCGGTTCTGTCATGTCG, ACCTGGTTCATCATCACTAATCAC, and probe #95. The reaction consisted of 50 cycles of denaturation for 30 s at 95°C, annealing for 30 s at 50°C, and extension for 30 s at 72°C. A standard curve was generated using a serial dilution of pooled sample of all single cDNAs. Expression of target genes was normalized using HPRT.

### 2.11. Bacterial Culture

The bacterial loads in tissues and blood were determined to investigate bacterial clearance under various treatment conditions to reach a better understanding of the delicate balance between enhancing the innate immune response and attenuating the inflammatory response. Lung tissue, liver tissue, and blood were examined for bacterial load. The tissues were homogenized in 3 ml NaCl for 1 minute before dilution to 5 ml with brain heart infusion (BHI) broth (Sigma-Aldrich, St. Louis, USA). For blood preparation, EDTA blood was used. Three concentrations (undiluted, 1 : 10, and 1 : 100) were prepared of the working solution using BHI. Ten *μ*l supernatant of tissue and blood dilutions were taken for bacterial culture in a blood agar-filled culture plate (Thermo, Waltham, US) to detect aerobic bacteria. Plates were incubated for 24 h at 37°C, and colonies were counted.

### 2.12. Statistical Analysis

All values were expressed as the mean ± SD. All statistical calculations were performed using SigmaStat (ver. 3.5.54; Systat Software GmbH, Erkrarth, Germany). The 72 h survival results were shown in a Kaplan-Meier curve, created using SigmaPlot 12. Groups of animals were compared employing the Student *t*-test in case of normal distribution of the data. If data were not normally distributed, the Mann–Whitney rank sum test was employed to compare sets of data in different animal groups. Data were expressed as the mean ± SD. *p* < 0.05 was considered statistically significant in this experiment.

## 3. Result

### 3.1. Partial Blockade of the Inflammatory Response Using Low-Dose LBPK95A Was Associated with Increased Survival Rate

Previous experiments revealed that G-CSF pretreatment improved the survival rate from 36% to 56% [14]. The survival rate was further increased by blocking the LPS-LBP interaction using high-dose LBPK95A (5 mg/kg) 2 h before septic insult [14]. However, application of the blocking peptide at the time of, or after, the septic insult is more important clinically. Therefore, we decided to apply the blocking peptide LBPK95A together with the feces slurry (0 h) in G-CSF-pretreated rats. Surprisingly, the combination treatment with application of high dose of LBPK95A at the time of septic insult (0 h) gave a significantly lower survival rate of only 10%. To elucidate the toxic effect of LBPK95A, the peptide was treated intraperitoneally, and a similar ALT level was observed 24 h after injection as in the control group (Figure S1). Based on these survival experiments, we speculated that the outcome of septic rats would be improved if LBP and bacterial/LPS load were in relative balance, prompting us to perform the dose-finding study.

**Figure 1:**
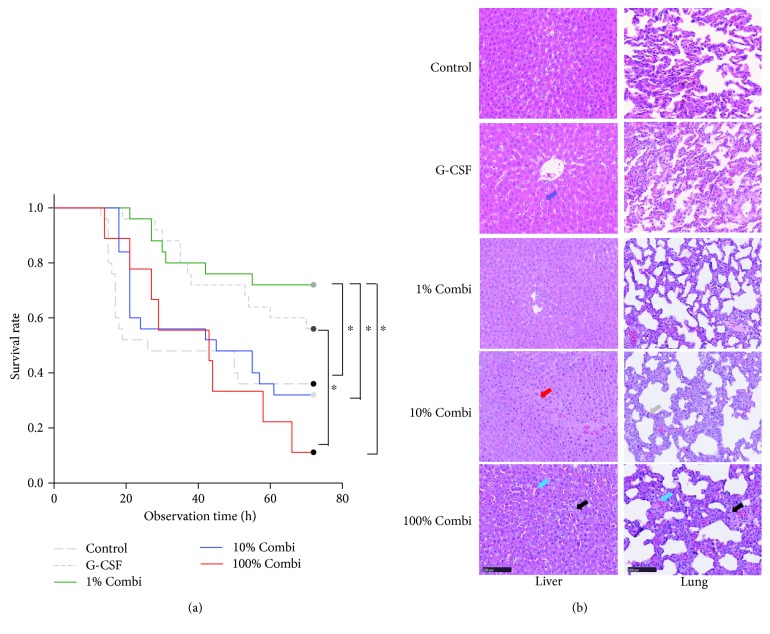
Modulation of inflammatory severity using various LBPK95A doses improved the therapeutic effect of G-CSF after septic insult. (a) Rats in the control group (control, injection of human stool suspension), G-CSF group (G-CSF, treatment with G-CSF for 5 days before septic insult), 1% Combi group (1% Combi group, G-CSF pretreatment for 5 days, 0.05 mg/kg LBPK95A injection immediately after injection of human stool suspension), 10% Combi group (10% Combi group G-CSF pretreatment for 5 days, 0.5 mg/kg LBPK95A injection immediately after injection of human stool suspension), and 100% Combi group (100% Combi group, G-CSF pretreatment for 5 days, 5 mg/kg LBPK95A injection immediately after injection of human stool suspension) were monitored every 3 h for 72 hours to record the activity and survival rate. ^∗^ *p* < 0.05. Gray line: data were collected from a previous study [14]. (b) Morphological evaluations of hepatic injury (left part) and kidney injury (right part) from all 5 groups at 12 h after septic insult. Hematoxylin and eosin staining, original magnification ×400. Black arrow: neutrophil infiltration, blue arrow: erythrocyte congestion, red arrow: hepatic necrosis, purple arrow: sinusoid dilatation, gray arrow: pulmonary edema. Hematoxylin and eosin staining, original magnification ×400.

As shown in Figure 1, when applying a lower dose of LBPK95A (10% Combi group, 0.5 mg/kg) at 0 h, the survival rate was 50%. Application of an even lower dose of LBPK95A (1% Combi group, 0.05 mg/kg) led to a 72 h survival rate of 70%.

### 3.2. Partially Blocking the Inflammatory Response Using Lower Doses of LBPK95A Decreased Tissue Damage

Organ damage is one of the leading causes of mortality of sepsis. The liver and the lung are particularly affected in most cases. Thus, we investigated the histomorphological alterations in liver and lung in the various groups to assess organ damage. The extent of liver and lung damage matched the survival data. As shown in Figure 1(b), liver injury, indicated by erythrocyte congestion, sinusoidal dilation and neutrophil infiltration were most severe in the control and 100% Combi groups at 12 h after infection. By comparison, only slight liver injury was observed in the 10% and 1% Combi groups.

**Figure 2:**
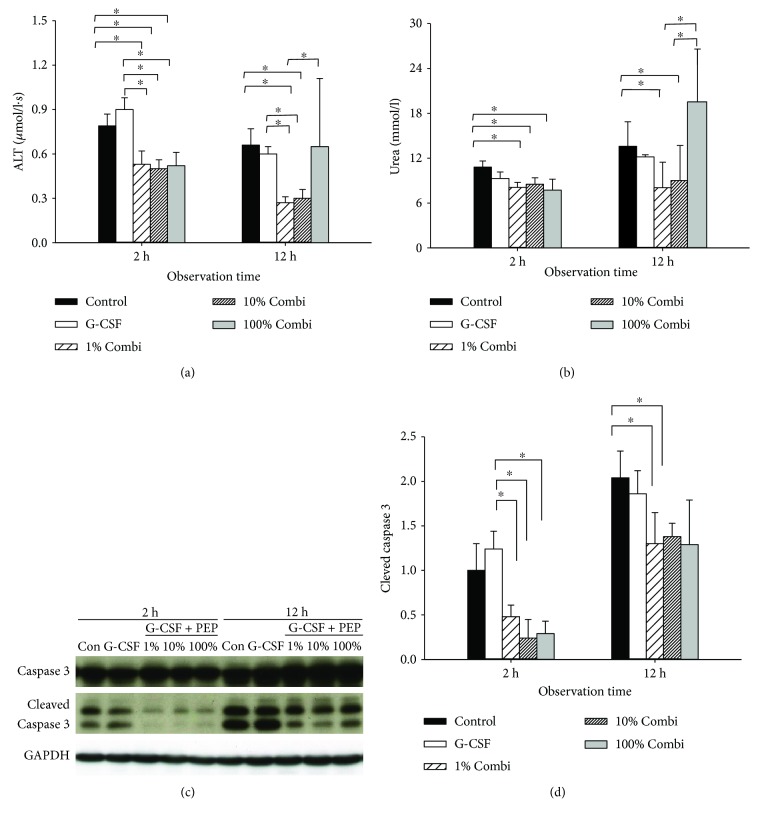
Partial blockade of the inflammatory response using low-dose LBPK95A decreased organ injury. (a) Serum ALT levels were measured to determine the severity of hepatic injury 2 h and 12 h after septic insult. (b) Serum urea levels were measured to determine the severity of kidney injury 2 h and 12 h after septic insult. (c) Hepatic levels of cleaved and total caspase 3 were measured by Western blot to determine apoptosis in liver tissue. (d) The gray value of bands was calculated by ImageJ. Data are shown as the mean ± SD. ^∗^ *p* < 0.05.

The release of liver enzymes also reflected the severity of hepatocellular injury. As shown in Figure 2(a), ALT levels in the combination groups were significantly lower after 2 h than in the control group and the G-CSF groups. Increased ALT levels were observed 12 h after septic insult in the 100% Combi group, but not in the 1% and 10% Combi groups. Kidney damage was evaluated by serum urea levels (Figure 2(b)). Kidney damage followed a similar pattern to that of liver damage. Urea levels in Combi treatment groups were lower than those of control groups 2 h after septic insult. By contrast, urea levels in the 100% Combi group were significantly higher than those of the 1% Combi and the 10% Combi groups. Of note, treatment with LBPK95A, irrespective of dose, resulted in significantly lower hepatic apoptosis than in control and G-CSF groups, 2 h and 12 h after septic insults. We examined caspase 3 cleavage to investigate apoptosis in the liver (Figure 2(c)). LBPK95A injection significantly lowered the expression of cleaved caspase 3 in all three Combi groups compared with those of the control group and the G-CSF group at 2 h and 12 h after septic insult.

### 3.3. Partial Blockade of the Inflammatory Response Using Low-Dose LBPK95A Decreased Neutrophil Infiltration

**Figure 3:**
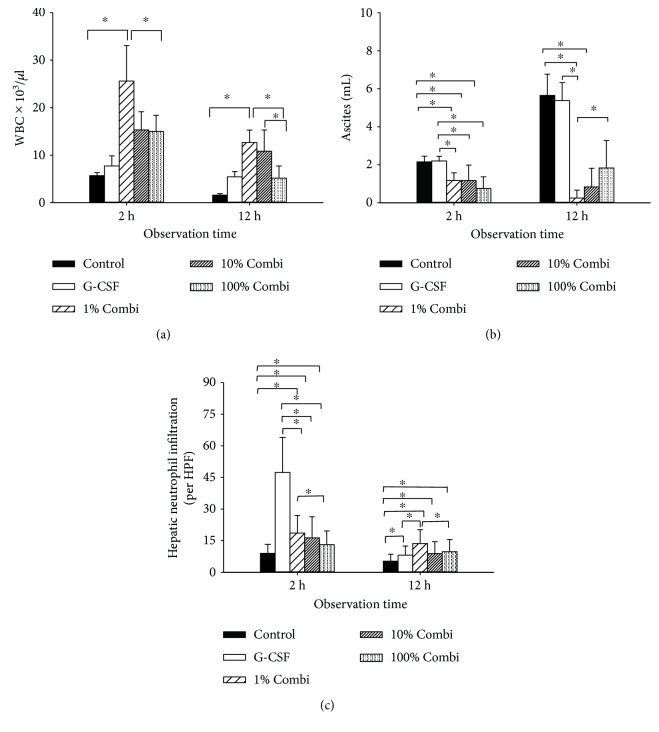
Partial blockade of the inflammatory response using low-dose LBPK95A decreased neutrophil infiltration to the site of inflammation. (a) WBC levels were measured in septic rats at 2 h and 12 h. (b) Volume of ascites was measured 2 h and 12 h after septic insult. (c) Hepatic neutrophil infiltration was assessed by performing ASDCL staining. Infiltrated cell numbers were counted at 2 h and 12 h after septic insult. ^∗^ *p* < 0.05.

To evaluate the function of innate immunity, levels of circulating WBC and hepatic neutrophil infiltration were assessed. G-CSF pretreatment improved neutrophil promotion and upregulation of LBP expression, as reported previously [16]. Counting the number of circulatory WBC confirmed that human G-CSF induced mobilization of WBC levels into the peripheral blood after septic insult (Figure 3(a)). G-CSF induced mobilized WBC infiltration to the liver within 2 h of septic insult, indicated by similar WBC levels compared to those of the control group, but with higher levels of ASDCL-positive cells infiltrating the liver. Of note, hepatic neutrophil infiltration in the G-CSF groups was significantly higher 2 h after septic insult, whereas the application of LBPK95A inhibited neutrophil infiltration in Combi groups. Hepatic neutrophils were increased 12 h after septic insult in the 1% Combi group, but not in the 10% and 100% Combi groups (Figure 3(b)). Ascites volume was used as an indicator of the severity of the local inflammatory response. The ascites volume in the G-CSF groups was significantly higher at 2 h than in the control group. By contrast, significantly less ascites was observed in the Combi groups 2 and 12 hours after septic insult (Figure 3(c)).

### 3.4. Partial Blockade of the Inflammatory Response Using Low-Dose LBPK95A Improved Bacterial Clearance

**Figure 4:**
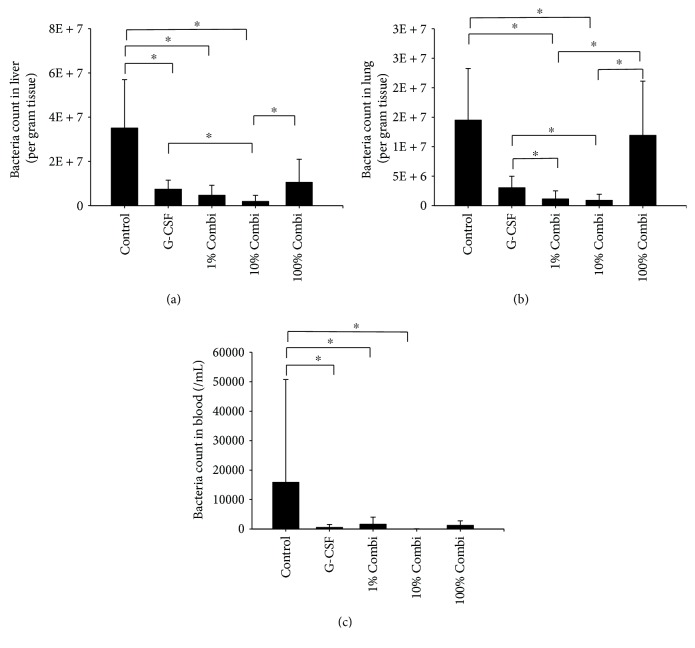
Partial decrease of the inflammatory response using low-dose LBPK95A promoted bacterial clearance after septic insult. Bacterial infiltration was observed in liver (a), lung (b), and blood (c) 2 h after septic insult. ^∗^ *p* < 0.05.

Bacterial clearance was assessed by tissue and blood culture (Figure 4). G-CSF pretreatment promoted bacterial clearance significantly, indicated by decreased bacterial counts in lung, liver, and blood. The bacterial count in the lung was further decreased upon treatment with LBPK95A in the 1% and 10% Combi groups, but not in the 100% Combi group. Decreased bacterial counts were also observed in liver tissue in the 1% and 10% Combi groups, but only the 10% Combi group reached statistical significance.

### 3.5. Partial Blockade of the Inflammatory Response Using Low-Dose LBPK95A Decreased Local and Systemic Inflammatory Responses

**Figure 5:**
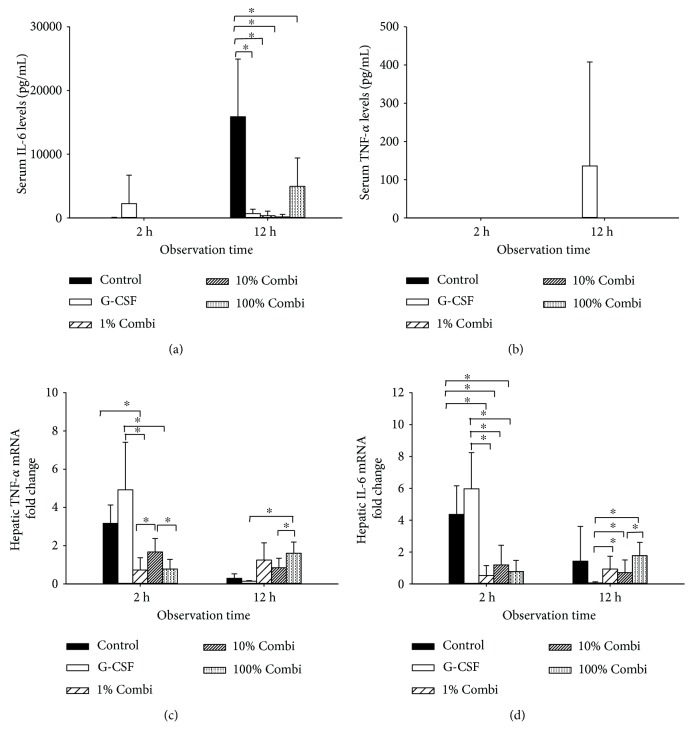
Partial decrease of the inflammatory response using low-dose LBPK95A decreased the inflammatory response. (a) Serum IL-6 levels were measured by ELISA 2 h and 12 h after the septic insult. (b) Serum TNF-*α* levels were measured using ELISA 2 h and 12 h after the septic insult. (c) Hepatic IL-6 mRNA expression was measured using qPCR 2 h and 12 h after septic insult. (d) Hepatic IL-6 mRNA expression was measured using qPCR 2 h and 12 h after septic insult. Data are shown as the mean ± SD. ^∗^ *p* < 0.05.

In this kinetic experiment, we measured serum levels of proinflammatory cytokines (TNF-alpha, IL-6) that are highly elevated in sepsis (Figures 5(a) and 5(b)). Serum TNF-alpha was only detectable in the G-CSF group 12 h after septic insult, whereas various levels of IL-6 were measured throughout the course of sepsis. In the control group, extremely high levels of IL-6 were observed after 12 h, reflecting the low survival rate in this untreated group. Compared with the control groups, significantly lower IL-6 levels were observed in the G-CSF group and in all three Combi groups (1%, 10%, and 100%).

Hepatic expression of TNF-a and IL-6 mRNA was assessed to explore the induction of local inflammatory responses (Figures 5(c) and 5(d)). G-CSF pretreatment caused inflammatory sensitization, indicated by increased hepatic TNF-a and IL-6 mRNA at both time points, 2 h and 12 h. Of note, treatment with LBPK95A decreased mRNA levels of TNF-a and IL-6 2 h after septic insult. However, an increase in cytokine expression was observed at 12 h.

**Figure 6:**
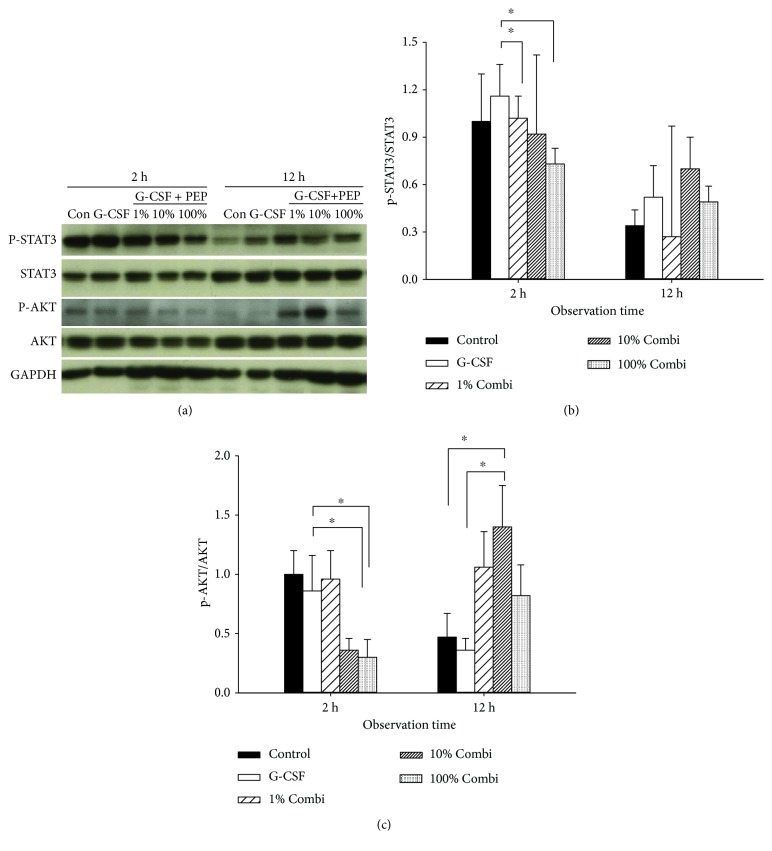
Partial decrease in the inflammatory response using low-dose LBPK95A caused delayed activation of STAT3 and AKT signal pathway. (a) Expression of p-STAT3, STAT-3, p-AKT, AKT, and GAPDH in liver tissue was measured by Western blot. (b, c) The gray value of bands: (b) p-STAT3/STAT3; (c) P/AKT/AKT) was calculated by ImageJ. Data are shown as the mean ± SD. ^∗^ *p* < 0.05.

The mRNA expression of inflammatory cytokines was associated with phosphorylation of STAT3 and AKT (Figure 6). The phosphorylation of STAT3 and AKT was significantly inhibited in all 3 Combi groups 2 h after septic insult but increased at 12 h. This accords with the observation of elevated mRNA levels of inflammatory cytokines.

## 4. Discussion

This study was designed to explore whether the dynamic balance between LBP-induced bacterial clearance and LBP-mediated LPS-sensitization was decisive for the therapeutic success of G-CSF-induced modulation of innate immunity in sepsis. We showed in immune-stimulated G-CSF-pretreated animals that blocking the LBP/LPS interaction with a low dose of blocking peptide (0.05 mg/kg) simultaneously with the feces suspension for sepsis induction was associated with a high survival rate. By contrast, increasing the dose of the blocking peptide in immune-stimulated animals to modulate the inflammatory response was associated with decreased survival rate, increased local neutrophil infiltration, and increased tissue damage.

G-CSF is needed for bacterial clearance. In vivo, monocytes and macrophages are major sources of G-CSF, particularly upon activation by pathogens, e.g., endotoxin and infection [18]. Bacterial infections result in transcription of the G-CSF gene and release of G-CSF that in turn accelerates the generation and functional activation of neutrophils. During acute infection, G-CSF is detected in the blood along with the release of proinflammatory cytokines [19, 20]. Administration of LPS also causes increased levels of circulating G-CSF [21]. Lack of G-CSF and subsequent neutropenia, e.g., induced by application of G-CSF depleting antibodies, was associated with a higher rate of sepsis in a dog model reported by Hammond et al. [22]. In line with their expectations, animals that had been made neutropenic by depletion of endogenous G-CSF were more susceptible to experimental peritonitis than were control animals, a finding consistent with the notion that neutrophils are indispensable for the host's defense against invading microorganisms [23, 24]. The same line of reasoning explains the effects of prophylactic G-CSF pretreatment: increasing the number of circulating neutrophils improved the outcome of septic animals. In our experiments, G-CSF-pretreated animals showed significantly higher numbers of neutrophils (Figure 3), longer survival times, and significantly higher survival rates than did the untreated group. This suggests that G-CSF and subsequent neutrophil upregulation led to increased bacterial clearance that improved outcomes.

However, conflicting effects of G-CSF were observed in septic patients and animal models. During the past few decades, there have been a variety of studies investigating the effect of G-CSF in patients with sepsis. However, consistent results have not been reported and no individual study has established whether G-CSF brings clinically important benefits for septic patients. Recently, Bo et al. demonstrated in a meta-analysis [8] that G-CSF treatment in patients with sepsis did not significantly reduce mortality at day 14 or day 28 and did not reduce in-hospital mortality. Similar results were reported by Stephens, who observed that addition of G-CSF to antibiotic therapy in newborn infants with suspected systemic infection did not significantly reduce mortality at 14 days or in-hospital mortality [25]. However, a trial showed that treatment with G-CSF in patients with septic shock was associated with dramatic improvement in patient survival [26]. By contrast, Quezado et al. reported that, in a sepsis model in canines, G-CSF administration as pretreatment therapy for long treatment times (96 h) did not improve the survival rate [27]. Preliminary clinical investigations confirmed these results. By contrast, several authors observed that G-CSF alone or in combination with antibiotics [11, 28] improved survival rates [29] in PCI or CLP models in rodents.

Our results may help to explain the conflicting results observed in clinical trials. The conflicting effects of G-CSF could be explained by the dynamic balance between innate immunity and the inflammatory response. The dynamic balance reflects the beneficial effects of G-CSF regarding bacterial clearance in relation to the detrimental adverse effects. In addition to upregulation of neutrophils, G-CSF leads to an increase in LBP [12].

LBP upregulation is associated with sensitization to LPS [16]. LBP is an acute phase protein that is upregulated in response to many stimuli (BDL [30], hemorrhagic shock [31], and infection [16]). What these stimuli have in common is that they put the patient at increased risk of undergoing sepsis. Our previous experiments showed that LBP upregulation leads to LPS sensitization [16], which is detrimental to the organism. On the other hand, LBP contributes to bacterial clearance [15]. In the present study, blockade of LBP using high doses of the blocking peptide (5 mg/kg LBPK95A as applied in the 100% Combi group) resulted in higher bacterial burden in tissues and blood than in the low-dose treatment group (0.05 mg/kg LBPK95A, in the 1% Combi group). Interestingly, we observed that the administration of LBPK95A (5 mg/kg) 6 hours after septic insult led to a low survival rate of rats (similar survival rate as control group, Figure S2). As the treatment of LBPK95A did not induce injury (Figure S1), we assume that treatment with the blocking peptide improved the outcome in sepsis but depended pivotally on the treatment time point [14]. The resulting idea was to take advantage of the beneficial effects of G-CSF and LBP in reducing bacterial burden, but to block the hyperinflammatory LBP-LPS response simultaneously. The LPS-induced inflammatory response could be decreased by reducing systemic LPS levels and by inhibition of the release of inflammatory cytokines.

Many strategies have been developed to decrease circulating endotoxin and to minimize inflammatory responses [32]. Direct neutralization of LPS using antibodies reduced systemic inflammatory responses. Zhang et al. [33] observed that delayed neutralization of IL-6 using an antibody reduced organ injury and decreased inflammatory cytokines in a hemorrhagic shock model. Analysis revealed that intravenous immunoglobulin reduced mortality in adults with sepsis. A meta-analysis by Qiu et al. indicated that anti-TNF agents gave a modest but significant decrease in the risk of death in sepsis patients [34]. However, the protective effects of either intravenous immunoglobulin or anti-TNF agent can hardly be proved in individual trials.

LBP blockade could be considered a novel strategy in modulation of inflammatory response in sepsis. Neutralization of LBP was accomplished by inhibition of the transfer of LBP-LPS complexes to the TLR4 complex to protect from induction of the inflammatory response [35]. Knapp et al. [36] reported that LBP (−/−) mice displayed decreased levels of early TNF-*α* and IL-6, reduced cytokine-induced neutrophil chemoattractant, reduced macrophage inflammatory protein production, and attenuated recruitment of polymorphonuclear leukocytes to the site of infection. However, depletion of LBP caused sensitization to *E. coli* infection, as LBP^−/−^ mice showed increased mortality, decreasing bacterial clearance and severe organ damage. This shows that LBP is necessary for bacterial elimination but is also detrimental due to sensitization to LPS. Araña et al. showed for the first time that interfering with the interaction between LPS and LBP using an LBP inhibitory peptide (LBPK95A) decreased inflammatory injury to animals [37]. Taken together, application of the blocking peptide could be a promising strategy to achieve the delicate balance between bacterial clearance and inflammatory activation. However, in our investigations, the effect of LBPK95A was not stable, especially in long-term tests, as LBPK95A is a short peptide (14 amino acids) and influenced by the half-life. We used to design LBPK95A-ZrP nanoparticles to prolong half life and increase the survival rate; however, the results were not promising (unpublished data). Therefore, selecting of a more powerful targeting system may be a key step in further investigation.

## 5. Conclusion

The beneficial effect of immune therapy can be attenuated by tissue damage caused by inflammation. In our study, the severity of inflammatory response was successfully downregulated using various doses of LBPK95A. However, it appears that a delicate balance between induction of innate immunity and blockade of inflammation is needed. These observations suggest that combining augmentation of innate immunity together with moderate blockade of the inflammatory response could be a novel strategy to treat sepsis.

## Data Availability

The data used to support the findings of this study are available from the corresponding author upon request.

## Supplementary Materials

Supplementary 1 Figure S1: the LBPK95A (5 mg/kg, intraperitoneal) treatment did increase the serum ALT levels 24 h after administration.

Supplementary 2 Figure S2: the administration of LBPK95A 6 h after septic insult did not improve the survival rate.

## References

[1] Hurley J. C., Guidet B., Offenstadt G., Maury E. (2012). Endotoxemia and mortality prediction in ICU and other settings: underlying risk and co-detection of gram negative bacteremia are confounders. *Critical Care*.

[2] Monneret G., Venet F., Pachot A., Lepape A. (2008). Monitoring immune dysfunctions in the septic patient: a new skin for the old ceremony. *Molecular Medicine*.

[3] Venet F., Monneret G. (2018). Advances in the understanding and treatment of sepsis-induced immunosuppression. *Nature Reviews Nephrology*.

[4] Fang H., Jiang W., Cheng J. (2015). Balancing innate immunity and inflammatory state via modulation of neutrophil function: a novel strategy to fight sepsis. *Journal of Immunology Research*.

[5] Wilkie-Grantham R. P., Magon N. J., Harwood D. T. (2015). Myeloperoxidase-dependent lipid peroxidation promotes the oxidative modification of cytosolic proteins in phagocytic neutrophils. *Journal of Biological Chemistry*.

[6] Winterbourn C. C., Kettle A. J. (2013). Redox reactions and microbial killing in the neutrophil phagosome. *Antioxidants & Redox Signaling*.

[7] Nauseef W. M. (2007). How human neutrophils kill and degrade microbes: an integrated view. *Immunological Reviews*.

[8] Bo L., Wang F., Zhu J., Li J., Deng X. (2011). Granulocyte-colony stimulating factor (G-CSF) and granulocyte-macrophage colony stimulating factor (GM-CSF) for sepsis: a meta-analysis. *Critical Care*.

[9] Hartung T., Von Aulock S., Schneider C., Faist E. (2003). How to leverage an endogenous immune defense mechanism: the example of granulocyte colony-stimulating factor. *Critical Care Medicine*.

[10] Gurleyik G., Yanikkaya G., Gurleyik E., Ozturk E., Dulundu E., Saglam A. (2007). Effects of granulocyte-colony stimulating factor on the polymorphonuclear leukocyte activity and the course of sepsis in rats with experimental peritonitis. *Surgery Today*.

[11] Bauhofer A., Lorenz W., Kohlert F., Torossian A. (2006). Granulocyte colony-stimulating factor prophylaxis improves survival and inflammation in a two-hit model of hemorrhage and sepsis. *Critical Care Medicine*.

[12] Ji Y., Dahmen U., Madrahimov N., Madrahimova F., Xing W., Dirsch O. (2009). G-CSF administration in a small-for-size liver model. *Journal of Investigative Surgery*.

[13] Schumann R. R. (2011). Old and new findings on lipopolysaccharide-binding protein: a soluble pattern-recognition molecule. *Biochemical Society Transactions*.

[14] Liu A., Weiss S., Fang H. (2015). Lipopolysaccharide-binding protein (LBP) blockade augments the protective effect of granulocyte colony-stimulating factor (G-CSF) in a rat sepsis model. *Shock*.

[15] Taddonio M. A., Dolgachev V., Bosmann M. (2015). Influence of lipopolysaccharide-binding protein on pulmonary inflammation in gram-negative pneumonia. *Shock*.

[16] Fang H., Liu A., Sun J., Kitz A., Dirsch O., Dahmen U. (2013). Granulocyte colony stimulating factor induces lipopolysaccharide (LPS) sensitization via upregulation of LPS binding protein in rat. *PLoS One*.

[17] Gonnert F. A., Recknagel P., Seidel M. (2011). Characteristics of clinical sepsis reflected in a reliable and reproducible rodent sepsis model. *Journal of Surgical Research*.

[18] Root R. K., Dale D. C. (1999). Granulocyte colony-stimulating factor and granulocyte-macrophage colony-stimulating factor: comparisons and potential for use in the treatment of infections in nonneutropenic patients. *The Journal of Infectious Diseases*.

[19] Cebon J., Layton J. E., Maher D., Morstyn G. (1994). Endogenous haemopoietic growth factors in neutropenia and infection. *British Journal of Haematology*.

[20] Kawakami M., Tsutsumi H., Kumakawa T. (1990). Levels of serum granulocyte colony-stimulating factor in patients with infections. *Blood*.

[21] da Silva A. M. T., Kaulbach H. C., Chuidian F. S., Lambert D. R., Suffredini A. F., Danner R. L. (1993). Shock and multiple-organ dysfunction after self-administration of Salmonella endotoxin. *The New England Journal of Medicine*.

[22] Hammond W. P., Csiba E., Canin A. (1991). Chronic neutropenia. A new canine model induced by human granulocyte colony-stimulating factor. *The Journal of Clinical Investigation*.

[23] Smith W. S., Sumnicht G. E., Sharpe R. W., Samuelson D., Millard F. E. (1995). Granulocyte colony-stimulating factor versus placebo in addition to penicillin G in a randomized blinded study of gram-negative pneumonia sepsis: analysis of survival and multisystem organ failure. *Blood*.

[24] Lieschke G. J., Grail D., Hodgson G. (1994). Mice lacking granulocyte colony-stimulating factor have chronic neutropenia, granulocyte and macrophage progenitor cell deficiency and impaired neutrophil mobilization. *Blood*.

[25] Carr R., Modi N., Doré C. J. (2003). G‐CSF and GM‐CSF for treating or preventing neonatal infections. *Cochrane Database of Systematic Reviews*.

[26] Stephens D. P., Fisher D. A., Currie B. J. (2002). An audit of the use of granulocyte colony-stimulating factor in septic shock. *Internal Medicine Journal*.

[27] Quezado Z., Parent C., Karzai W. (2001). Acute G-CSF therapy is not protective during lethal *E. coli* sepsis. *American Journal of Physiology-Regulatory, Integrative and Comparative Physiology*.

[28] Villa P., Shaklee C. L., Meazza C., Agnello D., Ghezzi P., Senaldi G. (1998). Granulocyte colony-stimulating factor and antibiotics in the prophylaxis of a murine model of polymicrobial peritonitis and sepsis. *The Journal of Infectious Diseases*.

[29] Lundblad R., Nesland J. M., Giercksky K. E. (1996). Granulocyte colony-stimulating factor improves survival rate and reduces concentrations of bacteria, endotoxin, tumor necrosis factor, and endothelin-1 in fulminant intra-abdominal sepsis in rats. *Critical Care Medicine*.

[30] Nishioji K., Sumida Y., Kamaguchi M. (2015). Prevalence of and risk factors for non-alcoholic fatty liver disease in a non-obese Japanese population, 2011–2012. *Journal of Gastroenterology*.

[31] Cotroneo T. M., Nemzek-Hamlin J. A., Bayliss J. M., Su G. L. (2012). Lipopolysaccharide binding protein inhibitory peptide alters hepatic inflammatory response post-hemorrhagic shock. *Innate Immunity*.

[32] Buttenschoen K., Radermacher P., Bracht H. (2010). Endotoxin elimination in sepsis: physiology and therapeutic application. *Langenbeck's Archives of Surgery*.

[33] Zhang Y., Zhang J., Korff S., Ayoob F., Vodovotz Y., Billiar T. R. (2014). Delayed neutralization of IL-6 reduces organ injury, selectively suppresses inflammatory mediator and partially normalizes immune dysfunction following trauma and hemorrhagic shock. *Shock*.

[34] Qiu P., Cui X., Sun J., Welsh J., Natanson C., Eichacker P. Q. (2013). Antitumor necrosis factor therapy is associated with improved survival in clinical sepsis trials: a meta-analysis. *Critical Care Medicine*.

[35] Kitchens R. L., Thompson P. A. (2005). Modulatory effects of sCD14 and LBP on LPS-host cell interactions. *Journal of Endotoxin Research*.

[36] Knapp S., de Vos A. F., Florquin S., Golenbock D. T., van der Poll T. (2003). Lipopolysaccharide binding protein is an essential component of the innate immune response to Escherichia coli peritonitis in mice. *Infection and Immunity*.

[37] Araña M. d. J., Vallespi M. G., Chinea G. (2003). Inhibition of LPS-responses by synthetic peptides derived from LBP associates with the ability of the peptides to block LBP-LPS interaction. *Journal of Endotoxin Research*.

