# Supplementary figures and images for: Modulation of Innate Immunity by G-CSF and Inflammatory Response by LBPK95A Improves the Outcome of Sepsis in a Rat Model
Source: J Immunol Res. 2018 Nov 7;2018:6085095. doi: 10.1155/2018/6085095 (PMC6247567; doi:10.1155/2018/6085095)

Figure S1

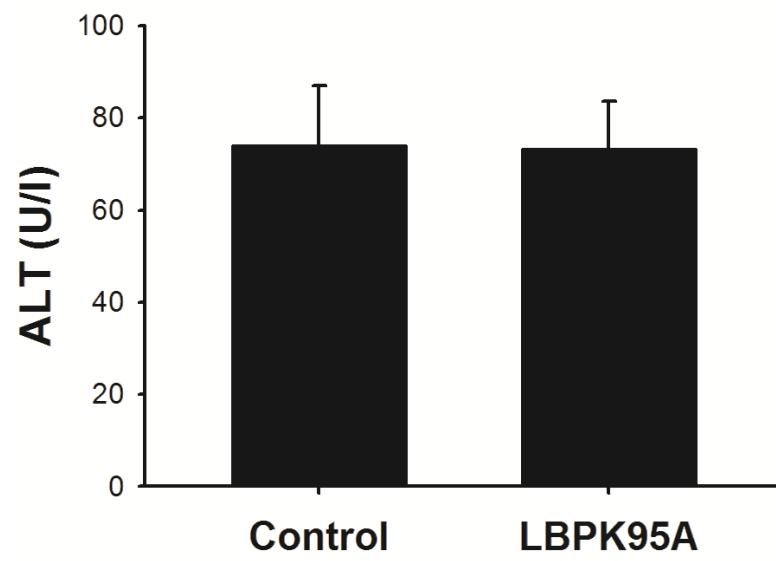

Supplement: Supplementary 1 — Figure S1: the LBPK95A (5 mg/kg, intraperitoneal) treatment did increase the serum ALT levels 24 h after administration. [file 6085095.f1.pdf]

Figure S2

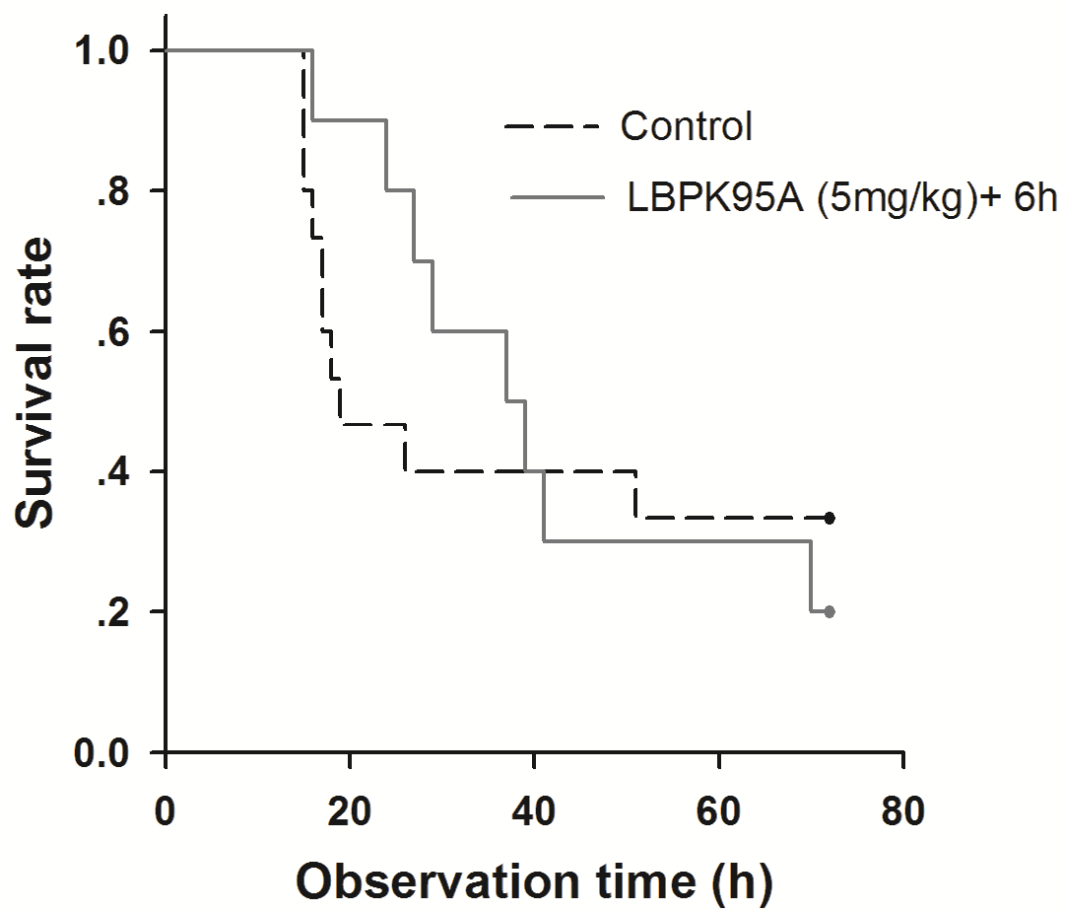

Supplement: Supplementary 2 — Figure S2: the administration of LBPK95A 6 h after septic insult did not improve the survival rate. [file 6085095.f2.pdf]
